# A physiologically based pharmacokinetic model for [^68^Ga]Ga-(HA-)DOTATATE to predict whole-body distribution and tumor sink effects in GEP-NET patients

**DOI:** 10.1186/s13550-023-00958-7

**Published:** 2023-02-03

**Authors:** Hinke Siebinga, Berlinda J. de Wit-van der Veen, Jos H. Beijnen, Thomas P. C. Dorlo, Alwin D. R. Huitema, Jeroen J. M. A. Hendrikx

**Affiliations:** 1 Department of Pharmacy and Pharmacology, The Netherlands Cancer Institute, Amsterdam, The Netherlands; 2 Department of Nuclear Medicine, The Netherlands Cancer Institute, Amsterdam, The Netherlands; 3 Department of Pharmacy, Uppsala University, Uppsala, Sweden; 4 Department of Clinical Pharmacy, University Medical Center Utrecht, Utrecht University, Utrecht, The Netherlands; 5 Department of Pharmacology, Princess Máxima Center for Pediatric Oncology, Utrecht, The Netherlands

**Keywords:** PBPK, ^68^Ga-DOTATATE, ^68^Ga-HA-DOTATATE, Whole-body distribution, Tumor sink effect

## Abstract

**Background:**

Little is known about parameters that have a relevant impact on (dis)similarities in biodistribution between various ^68^Ga-labeled somatostatin analogues. Additionally, the effect of tumor burden on organ uptake remains unclear. Therefore, the aim of this study was to describe and compare organ and tumor distribution of [^68^Ga]Ga-DOTATATE and [^68^Ga]Ga-HA-DOTATATE using a physiologically based pharmacokinetic (PBPK) model and to identify factors that might cause biodistribution and tumor uptake differences between both peptides. In addition, the effect of tumor burden on peptide biodistribution in gastroenteropancreatic (GEP) neuroendocrine tumor (NET) patients was assessed.

**Methods:**

A PBPK model was developed for [^68^Ga]Ga-(HA-)DOTATATE in GEP-NET patients. Three tumor compartments were added, representing primary tumor, liver metastases and other metastases. Furthermore, reactions describing receptor binding, internalization and recycling, renal clearance and intracellular degradation were added to the model. Scan data from GEP-NET patients were used for evaluation of model predictions. Simulations with increasing tumor volumes were performed to assess the tumor sink effect.

**Results:**

Data of 39 and 59 patients receiving [^68^Ga]Ga-DOTATATE and [^68^Ga]Ga-HA-DOTATATE, respectively, were included. Evaluations showed that the model adequately described image-based patient data and that different receptor affinities caused organ uptake dissimilarities between both peptides. Sensitivity analysis indicated that tumor blood flow and blood volume impacted tumor distribution most. Tumor sink predictions showed a decrease in spleen uptake with increasing tumor volume, which seemed clinically relevant for patients with total tumor volumes higher than ~ 550 mL.

**Conclusion:**

The developed PBPK model adequately predicted tumor and organ uptake for this GEP-NET population. Relevant organ uptake differences between [^68^Ga]Ga-DOTATATE and [^68^Ga]Ga-HA-DOTATATE were caused by different affinity profiles, while tumor uptake was mainly affected by tumor blood flow and blood volume. Furthermore, tumor sink predictions showed that for the majority of patients a tumor sink effect is not expected to be clinically relevant.

**Supplementary Information:**

The online version contains supplementary material available at 10.1186/s13550-023-00958-7.

## Introduction

The incidence of neuroendocrine tumors (NETs) has increased over the last decades, which is mainly due to improved awareness and better diagnostic imaging using radiolabeled somatostatin analogues (SSAs) [1, 2]. The relatively new theranostics concept encompasses diagnosis, treatment and therapy evaluation with the same radiolabeled peptide. For radionuclide therapy with Lutetium-177 (^177^Lu)-labeled SSAs, DOTA-Tyr^3^-octreotate (DOTATATE) is nowadays used most often as it is incorporated in the FDA- and EMA-approved Lutathera^®^ [3, 4]. On the other hand, some Gallium-68 (^68^Ga)-labeled SSAs are FDA -and EMA-approved and several are currently used for routine imaging of NETs, such as DOTA-Tyr^3^-octreotide (DOTATOC), DOTA-1-NaI^3^-octreotide (DOTANOC), DOTATATE and DOTA-iodo-Tyr^3^-octreotate (HA-DOTATATE; HA, high-affinity) [5–8]. Distribution patterns of these ^68^Ga-labeled SSAs have been assessed clinically, comparing observed uptake differences and similarities for DOTATOC, DOTANOC and HA-DOTATATE with DOTATATE [5, 9–12]. There appears no clear preference of one compound over the others, so choices for a diagnostic peptide are primarily driven by local availability.

Although DOTATATE and its twin HA-DOTATATE should show similar uptake profiles on diagnostic imaging [5, 13], relevant variations in uptake patterns were observed in certain patients in clinical practice. Mismatches in biodistribution and tumor uptake have also been described between pre-therapeutic imaging and actual (^177^Lu-)therapy [14–19]. As these differences in biodistribution and tumor uptake limit the predictive value of pre-treatment imaging, it is important to identify chemical, biological or physiological factors (i.e., compound- or system-specific parameters) that may influence peptide accumulation.

Physiologically based pharmacokinetic (PBPK) models have been introduced as a tool to mathematically describe and predict whole-body distributions of (radio)pharmaceuticals [20]. The application of these models in the field of nuclear medicine is still limited, but these models may help to give an insight in factors that cause inter-individual uptake variability and dissimilarities in distribution profiles between the different SSAs. Only a few PBPK models have been developed for the various SSAs [21–25], but, to our knowledge, direct comparisons of distribution profiles for different SSAs have not been performed yet. Recently, a PBPK model has been developed by our group describing normal organ distribution in patients without NETs, which showed the importance of somatostatin receptor 2 (SSTR2) density and administered peptide amount on the organ distribution for [^68^Ga]Ga-DOTATATE [26]. Extending this model with tumor compartments will allow prediction of organ and tumor peptide accumulation in patients with different (tumor) physiologies. Such an approach has previously been published for [^177^Lu]Lu-DOTATATE [27]. However, this [^177^Lu]Lu-DOTATATE model with varying tumor volumes was developed in virtual patients; thus, no comparisons with (many) clinical data were performed. Additionally, this study did not assess the effect of increasing tumor burden on organ uptake, even though a high total tumor burden could result in reduced organ uptake, also known as the tumor sink effect [28]. This sequestration of the radiotracer into large-volume metastasis is known to have a direct effect on biodistribution and can, in diagnostic imaging, lead to deviating patterns in normal distributions and reduced uptake in reference tissues. In radionuclide therapy, this phenomenon is even more relevant as it may lead to a dependency between absorbed organ dose and tumor volume for fixed peptide amounts.

Therefore, the aim of this current study was to develop a PBPK model to describe and compare normal organ and tumor distribution of both [^68^Ga]Ga-DOTATATE and [^68^Ga]Ga-HA-DOTATATE and to identify factors that might cause biodistribution and tumor uptake differences between both peptides. Furthermore, the relevance of the tumor sink effect in patients with gastroenteropancreatic (GEP) NETs was assessed.

## Methods

### Patient population and imaging data

Patients diagnosed with GEP-NET who received a whole-body [^68^Ga]Ga-DOTATATE or [^68^Ga]Ga-HA-DOTATATE PET/CT between August 2011 and April 2016 were included. Patients without apparent NET-lesions on diagnostic PET/CT were allocated to a separate cohort that was used as a control group, which represented patients that were referred with suspected NET or received treatment for prior NET. Informed consent was obtained via institutional procedures from all individual participants included in the study.

Patients were treated according to the EANM-guidelines [29], and SSA therapy was withdrawn prior to [^68^Ga]Ga-(HA-)DOTATATE administration (where (HA-)DOTATATE refers to both peptides). ^68^Ga-labeled (HA-)DOTATATE was produced in-house according to validated procedures described previously [30]. An intravenous injection of approximately 100 MBq ^68^Ga-labeled (HA-)DOTATATE was administered, and at ~ 45 min post-injection a scan was acquired on a Gemini TF PET/CT (Philips, the Netherlands) with 2–2.5 min per bed position from skull to thighs (axial field of view of 18 cm). Image data was reconstructed using the Philips specific BLOB OS-ToF algorithm with ‘normal’ smoothing (isotropic 4 mm voxels).

SUV_peak_, SUV_max_ and radioactivity concentrations (MBq/L) for aorta, spleen, thyroid, liver, primary tumor and metastatic lesions > 2 cm in diameter were determined by placing spherical volume of interests (VOIs) of at least 2 cm. Then, all radioactivity concentrations were corrected for decay to time of injection and subsequent peptide concentrations (µg/L) per organ or tumor were calculated based on the measured radioactivity concentrations and the administered specific activities (MBq/µg).

To assess the effect of tumor burden on biodistribution, patients were divided into three groups based on quantity and location of metastases on their clinical PET/CT report. These three groups were: (1) patients with primary tumors or with ≤ 2 metastases (limited disease), (2) patients with liver-only metastases, and (3) patients with extensive metastases (i.e. , not classified as group 1 or 2). The PBPK model predictions, for both [^68^Ga]Ga-DOTATATE and [^68^Ga]Ga-HA-DOTATATE, were evaluated with clinical imaging data for these three groups separately.

### PBPK model development

A PBPK model for GEP-NET patients was developed in PK-Sim^®^ and MoBi^®^ (Open Systems Pharmacology software, version 9.0) using the standardized protein base model [31]. Compound-specific information includes the physicochemical parameters of the compound, and this was separately added for DOTATATE and HA-DOTATATE to obtain compound-specific predictions. Eventually, predictions were performed separately for each patient group, based on metastatic status, for both peptides.

All input parameters for peptide specific distribution and uptake, including intracellular degradation rates and SSTR2 amounts, were based on the previous described [^68^Ga]Ga-DOTATATE PBPK model [26]. Compound-specific parameters for DOTATATE were also derived from the previous published PBPK model [26], while compound-specific input parameters of HA-DOTATATE (molecular weight and lipophilicity) were based on literature. The input parameters molecular weight and lipophilicity for [^68^Ga]Ga-HA-DOTATATE were 1628.5 g/mol and −3.12, respectively [32]. Plasma protein binding for [^68^Ga]Ga-HA-DOTATATE was unknown, but a major difference compared to [^68^Ga]Ga-DOTATATE was not expected; thus, the fraction unbound was set at 0.69, accordingly. Renal clearance was manually scaled to a predicted 13% unchanged excretion in urine within the first 2 h post-injection for all patients [33]. For the predictions per group for both peptides, the mean estimated glomerular filtration rate (GFR) per group was used as renal function input (calculated using the Cockcroft–Gault equation [34]). In addition, all other assessed patient characteristics (i.e., age, weight and height) were used as system-specific input parameters. Internalization rates were assumed to be similar for organ and tumor tissue and were fixed to 0.268 min^−1^. This was based on a 1.67-fold increase in internalization rate for [^68^Ga]Ga-HA-DOTATATE compared to [^68^Ga]Ga-DOTATATE (accumulation of [^68^Ga]Ga-HA-DOTATATE in spleen plateaued 30 min post-injection compared to 50 min post-injection for [^68^Ga]Ga-DOTATATE) [26, 33, 35]. Unknown input parameters for HA-DOTATATE (the equilibrium dissociation constant (*K*_D_) and dissociation rate constant (*k*_off_)) were optimized by using all observed spleen concentrations from all included patients at once and thus optimized values represent *K*_D_ and *k*_off_ for the entire population. By using observed data of spleen, which expresses SSTR2 but not SSTR5 [32, 36], it was ensured that the optimized affinity values of HA-DOTATATE were specific for SSTR2. Model fitting was performed using a built-in Monte Carlo algorithm for parameter identification to optimize selected input parameter to describe the data best.

Separate tumor compartments were added to the model to describe distribution to these compartments and the effect of this tumor uptake on normal organ uptake. Since physiological tumor characteristics can differ between primary tumors and its metastases, three compartments were added: primary tumor, liver metastases and other metastases. All metastases other than liver metastases were gathered in one compartment since only small uptake differences were observed in clinical practice between those metastases and also for reasons of model simplicity. Tumor physiology characteristics for all three tumor compartments were based on literature. The tumor volume for the primary tumor was set at 10 mL for all groups, and liver metastases were assumed to have a total volume of 50 mL. Volumes of other tumor metastases differed for each patient group and were set at 5 mL, 0 mL and 50 mL for groups 1, 2 and 3, respectively, based on clinical observations indicating a mean total tumor volume of approximately 65 mL in 232 patients [37]. Although quantification of tumor volumes in this article was debatable, since there is no gold standard for tumor volume measurements, yet a mean total NET volume of approximately 65 mL reflected the population of patients treated in our hospital. Besides, this was comparable to median tumor volumes that were used (or fitted) in previous PBPK models [21, 27, 38, 39]. Vascular sub-compartments within tumors were estimated based on literature values for primary tumors and liver metastases, resulting in fraction vascular of 0.21 and 0.17, respectively [40–45]. Fraction vascular for other metastases was unknown and fixed to 0.075 based on previous PBPK models [21, 22, 46]. The interstitial fraction was assumed to be similar for each tumor and was fixed to 0.3 [22]. Blood flow was set at 152 mL/min/100 g for the primary tumor compartment [45]. In absence of relevant data for distant metastatic sites, the same value was used for these sites. For liver metastases, a higher blood flow of 203 mL/min/100 g was used as an input parameter, due to their nature of hyperperfusion [42–44]. SSTRs were added to the tumor compartments with an expression relative to spleen. Since NET metastases showed higher SSTR2 expressions compared to primary tumor sites [47], a higher relative expression was added to this compartment (relative value of 1.5 for primary tumors and 2 for all metastases). These fractional differences between tumors compared to spleen were in accordance with previous NET PBPK models [21, 22]. Transcapillary exchange of the radiopharmaceutical from plasma to the interstitial space was described by the two-pore formalism [46]. Pore radii for small and large pores were fixed to 4.5 and 500 nm, respectively, representing a leaky tumor vessel capillary [46, 48]. The flow fraction via the large pores was 0.8, reflecting a discontinuous membrane as expected in tumors [46]. Hydraulic conductivity describes passage of porous material and was fixed to a literature value of 0.00126 mL/N/min for tumors [49, 50].

Minimum and maximum ranges for administered peptide amounts per group and SSTR2 organ expression (derived from the previously published PBPK model) were imputed to derive prediction intervals for organ and tumor radionuclide distributions [26]. In addition, to take into account a high variability in tumor uptake, prediction intervals in all three tumor compartments were based on observed (inter)quartile ranges in blood flow and blood volumes of the specific compartment [42–45]. For other metastases, blood flow was assumed to be comparable to primary tumors and fraction vascular was assumed to vary from 50 to 150% relative to the median. This resulted in minimum and maximum blood flow of 53 and 252 mL/min/100 g for primary tumors and other metastases and 139 and 363 mL/min/100 g for liver metastases. Fraction vascular ranged from 0.09 to 0.34 for primary tumors, 0.1 to 0.23 for liver metastases and 0.025 to 0.125 for other metastases.

A sensitivity analysis was performed to calculate the sensitivity of the PK model output for certain parameter assumptions using a build-in algorithm, which was performed by alteration of input parameters with ± 10% [51]. This sensitivity (*S*_*i,j*_) is calculated using the following equation:

1 $$S_{i,j} = \frac{{\Delta {\text{PK}}_{j} }}{{\Delta p_{i} }}*\frac{{p_{j} }}{{{\text{PK}}_{j} }}$$

where *PK*_*j*_ is a PK parameter of a certain output to an input parameter (*p*_*j*_)_._ Thus, the sensitivity for the PK parameter to that input parameter was calculated as the ratio of the relative change of that PK parameter (*ΔPK*_*j*_) and the relative variation of the input parameter (*Δp*_*i*_). A sensitivity value of − 1 implies that a 10% increase of the input parameter resulted in a 10% decrease of the PK parameter output.

### DOTATATE versus HA-DOTATATE

Administered patient doses of [^68^Ga]Ga-DOTATATE and [^68^Ga]Ga-HA-DOTATATE are based on radioactivity (~ 100 MBq per administration). Specific activities (MBq/µg) were derived from the tracer production logs to calculate the administered absolute peptide amount. However, specific activities, and thus administered peptide amounts, differed between production batches and peptides. In general, this resulted in higher administered total peptide amounts for DOTATATE compared to HA-DOTATATE.

It is of interest to directly compare organ uptake between both peptides to assess (dis)similarities in this organ accumulation, without discrepancies in administered peptide amounts that could alter such a comparison. Due to a change in production procedures in 2017, an additional group of GEP-NET patients receiving higher peptide amounts of [^68^Ga]Ga-HA-DOTATATE (comparable to administered DOTATATE amounts) could be selected for a subanalysis to exclude a potential effect of different administered peptide amounts for [^68^Ga]Ga-DOTATATE and [^68^Ga]Ga-HA-DOTATATE on organ uptake. The sample size of this additional group was matched to the included patients for PBPK evaluation receiving HA-DOTATATE, and spleen was used as a reference organ to compare uptake (SUV_peak_ and SUV_max_).

### Tumor sink effect

Imaging data regarding uptake (SUV_peak_ and SUV_max_) were compared between the three patient groups (limited disease, liver-only and extensive metastases) to assess differences in organ and tumor uptake for [^68^Ga]Ga-DOTATATE and [^68^Ga]Ga-HA-DOTATATE, potentially revealing a tumor sink effect. Organ uptake in GEP-NET patients was compared to the control group, to reveal any effect of tumor load and location on normal organ uptake (spleen, liver and thyroid).

Predictions to identify a relevant tumor sink effect for [^68^Ga]Ga-DOTATATE and [^68^Ga]Ga-HA-DOTATATE were performed using the final PBPK model. This was performed by manually increasing tumor volumes of all separate tumor compartments and comparing the accumulation plateau (or C_max_) in spleen as a reference organ.

### Statistical tests

Differences between patient characteristics and demographics receiving DOTATATE and HA-DOTATATE were evaluated using a Student’s t-test (continuous variables), a Wilcoxon test (continuous variables in case of unequal variances) or a Chi-squared test (categorical variables). An ANOVA test was used to compare patient characteristics of the patient groups for both DOTATATE and HA-DOTATATE. Kruskal–Wallis tests were performed to compare uptake in organs and tumors (SUV_max_ and SUV_peak_) between patient groups, where a Bonferroni correction was applied to account for multiple testing when testing which groups differed significantly from each other. Statistical tests were performed in R (version 3.6.3) [52]. Continuous variables are displayed as mean ± standard deviation (range). A *p*-value less than 0.05 was considered statistically significant.

## Results

### Patient data

One patient was excluded from this analysis, because this patient erroneously received an exceptionally high DOTATATE amount (29.8 µg). This resulted in a final inclusion of 39 and 59 GEP-NET patients receiving either [^68^Ga]Ga-DOTATATE or [^68^Ga]Ga-HA-DOTATATE, respectively. The peptide amounts and administered radioactivity were 13.1 ± 2.18 µg (7.16–16.6 µg) and 85.8 ± 16.0 MBq (50.3–132.7 MBq) for [^68^Ga]Ga-DOTATATE, and 6.06 ± 2.26 µg (2.43–11.1 µg) and 85.7 ± 14.1 MBq (43.8–106.3 MBq) for [^68^Ga]Ga-HA-DOTATATE. Mean injection-acquisition interval was 44 ± 6 min (34–55 min) for [^68^Ga]Ga-DOTATATE and 44 ± 6 min (29–55 min) for [^68^Ga]Ga-HA-DOTATATE. Patients were classified based on disease extent as limited disease, liver-only or extensive metastases. In addition, 43 and 45 control subjects receiving [^68^Ga]Ga-DOTATATE and [^68^Ga]Ga-HA-DOTATATE, respectively, were included. All patient characteristics, demographics and imaging details are shown in Tables 1 and 2; details for the subanalysis regarding patients receiving higher peptide amounts of [^68^Ga]Ga-HA-DOTATATE are displayed in Additional file 1: Table S1.

**Table 1:** Patient demographics and characteristics for all GEP-NET patients receiving [^68^Ga]Ga-DOTATATE or [^68^Ga]Ga-HA-DOTATATE

	[^68^Ga]Ga-DOTATATE (*n* = 39)	[^68^Ga]Ga-HA-DOTATATE (*n* = 59)	*p*-value
Sex			
Male	14 (36%)	31 (53%)	0.356
Female	25 (64%)	28 (47%)	
Age (years)	62 ± 11.6 (39–84)	62 ± 10.8 (40–82)	0.895
Weight (kg)	77.5 ± 14.7 (53.0–117)	72.0 ± 13.8 (44.0–113)	0.0697
Height (cm)	171 ± 7.62 (157–185)	173 ± 8.25 (158–192)	0.290
GFR (Cockcroft Gault, mL/min)	90.7 ± 26.9 (33.8–147)	82.3 ± 25.2 (35.8–183)	0.149
Peptide amount administered (µg)	13.1 ± 2.18 (7.16–16.6)	6.06 ± 2.26 (2.43–11.1)	< 0.001
Radioactivity administered (MBq)	85.8 ± 16.0 (50.3–133)	85.7 ± 14.1 (43.8–106)	0.981
Injection-acquisition interval (min)	44 ± 6 (34–55)	44 ± 6 (29–55)	0.952
Primary tumor			
Pancreas	9 (23%)	17 (29%)	0.815
Small intestine	23 (59%)	34 (58%)	
Colon	4 (10%)	4 (7%)	
Rectum	–	1 (2%)	
Stomach	3 (8%)	3 (5%)	
Tumor grade			
1	21 (54%)	20 (34%)	0.103
2	12 (31%)	27 (46%)	
Unknown	6 (15%)	12 (20%)	
SUV_max_			
Aorta	1.81 ± 0.635 (0.93–3.26)	1.25 ± 0.408 (0.62–2.36)	< 0.001
Spleen	18.2 ± 6.14 (7.07–31.5)	27.7 ± 9.04 (12.6–52.8)	< 0.001
Liver	7.47 ± 2.83 (2.81–17.7)	11.3 ± 2.92 (5.23–18.4)	< 0.001
Thyroid	3.47 ± 1.45 (1.38–7.85)	5.30 ± 2.17 (2.17–14.9)	< 0.001
Primary tumor	24.1 ± 15.3 (6.33–63.5)	29.9 ± 18.6 (8.58–74.9)	0.291
Liver metastases	25.1 ± 12.4 (6.97–53.7)	33.1 ± 13.2 (13.8–79.8)	0.014
Other metastases	15.5 ± 13.3 (2.12–83.4)	21.3 ± 15.8 (2.71–75.7)	0.020
SUV_peak_			
Aorta	1.30 ± 0.425 (0.64–2.33)	0.918 ± 0.278 (0.52–1.75)	< 0.001
Spleen	16.4 ± 5.52 (6.34–26.3)	25.2 ± 7.91 (11.5–41.2)	< 0.001
Liver	6.07 ± 2.19 (2.45–12.9)	9.86 ± 2.63 (4.44–16.7)	< 0.001
Thyroid	2.52 ± 1.18 (0.80–6.50)	3.81 ± 1.50 (1.41–9.54)	< 0.001
Primary tumor	18.0 ± 13.1 (4.74–53.7)	20.1 ± 11.9 (6.21–48.7)	0.509
Liver metastases	19.6 ± 10.3 (6.18–47.2)	25.4 ± 10.1 (11.5–54.6)	0.017
Other metastases	9.67 ± 8.44 (1.06–44.5)	14.6 ± 11.5 (1.53–51.5)	0.007
Peptide accumulation (µg/L)			
Spleen	2.81 ± 1.10 (1.06–4.98)	2.14 ± 1.01 (0.66–5.29)	0.002
Primary tumor	2.99 ± 2.50 (0.77–8.58)	1.81 ± 1.64 (0.31–7.67)	0.092
Liver metastases	3.48 ± 2.00 (0.76–8.39)	2.30 ± 1.09 (0.85–4.88)	0.009
Other metastases	1.34 ± 1.13 (0.19–6.36)	1.05 ± 0.90 (0.11–3.59)	0.067

Continuous variables are shown as mean ± standard deviation (range) and categorical variables as number (%)

*GFR* Glomerular filtration rate; *SUV* Standardized uptake value

**Table 2:** Patient characteristics of the four patient groups, representing control group: patients without NET diagnosis based on PET/CT, group 1: primary tumors solely or with a few (≤ 2) metastases (limited disease), group 2: liver-only metastases and group 3: extensive metastases

Parameters	[^68^Ga]Ga-DOTATATE					[^68^Ga]Ga-HA-DOTATATE				
	Control group (*n* = 41)	Group 1 (*n* = 10)	Group 2 (*n* = 4)	Group 3 (*n* = 25)	*p*-value	Control group (*n* = 45)	Group 1 (*n* = 21)	Group 2 (*n* = 7)	Group 3 (*n* = 31)	*p*-value
Male	22 (54%)	4 (40%)	2 (50%)	8 (32%)	< 0.001	20 (44%)	10 (48%)	3 (43%)	15 (48%)	< 0.001
Female	19 (46%)	6 (60%)	2 (50%)	17 (68%)		25 (56%)	11 (52%)	4 (57%)	16 (52%)	
Age (years)	56 ± 14 (22–79)	64 ± 9 (46–75)	57 ± 12 (48–75)	62 ± 13 (39–84)	0.200	59 ± 12 (27–84)	65 ± 9.83 (47–80)	57 ± 9.81 (44–67)	61 ± 11.7 (40–82)	0.282
Weight (kg)	81.1 ± 18.4 (53–120)	77.6 ± 14.2 (60–104)	88.3 ± 13.0 (73–103)	75.7 ± 15.0 (53–117)	0.415	87.6 ± 22.3 (40–145)	73.5 ± 16.1 (44–113)	78.6 ± 18.1 (52–102)	69.6 ± 10.6 (51–100)	0.001
Height (cm)	176 ± 11.3 (155–196)	169 ± 6.72 (158–180)	174 ± 9.29 (164–182)	171 ± 7.94 (157–185)	0.270	173 ± 10.2 (154–193)	171 ± 8.30 (160–186)	174 ± 11.3 (159–185)	174 ± 7.70 (158–192)	0.871
GFR (Cockcroft Gault, mL/min)	94.8 ± 31.5 (49.6–160)	101 ± 31.5 (47.4–146)	99.6 ± 10.2 (93.4–115)	85.3 ± 26.5 (33.8–124)	0.482	115 ± 42.5 (41.7–190)	84.0 ± 21.5 (50.3–117)	93.0 ± 49.1 (41.3–183)	79.3 ± 20.4 (35.8–123)	0.107
Peptide amount administered (µg)	12.5 ± 2.65 (8.05–16.9)	13.1 ± 1.45 (10.6–15.7)	14.5 ± 1.08 (13.4–16.0)	12.8 ± 2.48 (7.16–16.6)	0.465	6.13 ± 2.29 (2.49–11.2)	5.22 ± 1.58 (2.43–7.78)	6.74 ± 2.72 (3.31–11.1)	6.47 ± 2.44 (3.37–11.0)	0.204
Radioactivity administered (MBq)	91.7 ± 23.4 (43.4–130)	79.7 ± 14.2 (57.8–101)	83.7 ± 15.3 (64.7–99.6)	88.5 ± 16.6 (50.3–133)	0.378	89.5 ± 11.4 (66.0–110)	84.9 ± 14.2 (60.1–106)	85.8 ± 12.0 (69.0–105)	86.2 ± 14.8 (43.8–106)	0.513
Injection-acquisition interval (min)	48 ± 11 (35–78)	43 ± 7 (34 -55)	43 ± 2 (41–45)	44 ± 6 (35–55)	0.153	45 ± 6 (35–55)	42 ± 6 (29–55)	44 ± 7 (35–52)	45 ± 6 (35–55)	0.358

Continuous variables are shown as mean ± standard deviation (range) and categorical variables as number (%)

*GFR* Glomerular filtration rate

### PBPK model predictions

**Fig. 1:**
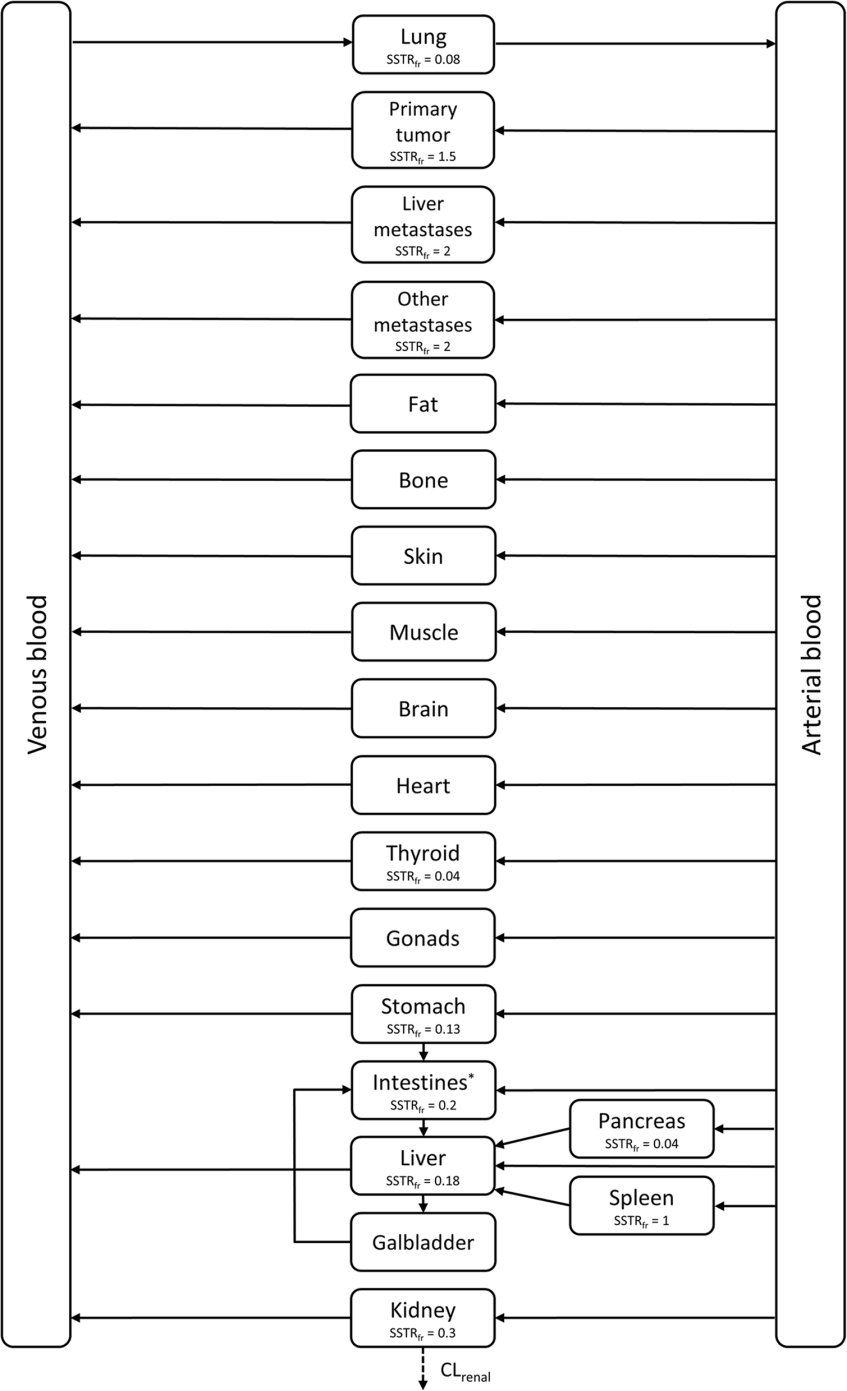
Structural overview of the multi-compartment PBPK model for GEP-NET patients, where somatostatin receptor expression fractions (related to spleen) are depicted as SSTR_fr_. *Intestinal compartment consists of small intestine (SSTR2 fraction = 0.09) and large intestine (SSTR2 fraction = 0.11). CL_renal_: renal clearance; SSTR_fr_: somatostatin receptor fraction

**Fig. 2:**
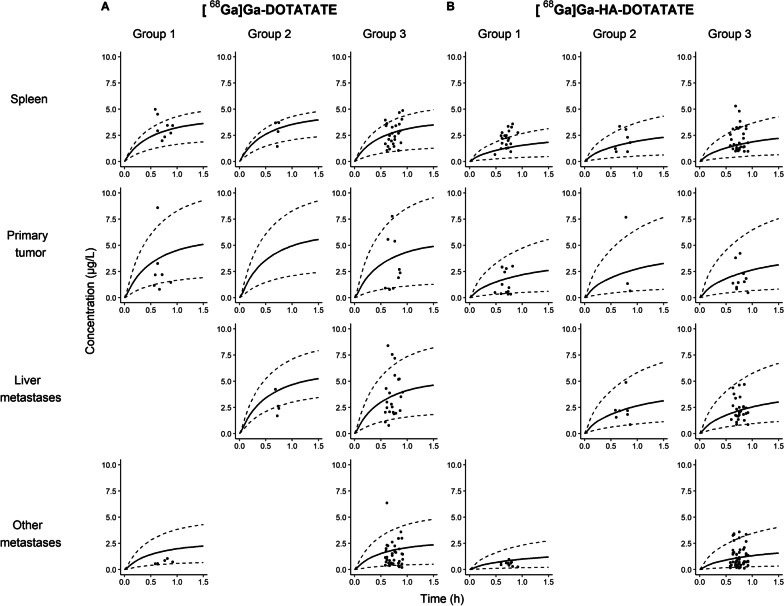
PBPK model prediction results per group (solid lines) for [^68^Ga]Ga-DOTATATE (**A**) and [^68^Ga]Ga-HA-DOTATATE (**B**), including minimum and maximum population ranges (dashed lines) that were based on administered peptide amount ranges, SSTR2 variability in organs, tumor blood flow variability and tumor blood volume variability. Missing data in group 2 for [^68^Ga]Ga-DOTATATE primary tumor uptake due to resection of the primary tumor for all patients (*n* = 4)

An overview of the PBPK model structure is provided in Fig. 1. Optimized values for HA-DOTATATE were 0.227 nmol/L for *K*_D_ and 0.00709 min^−1^ for *k*_off_. An overview of compound-specific input parameters for both peptides and main system-specific input parameters for all tumor compartments is shown in Table 3. Final simulated concentration–time curves for the patient groups for both [^68^Ga]Ga-DOTATATE and [^68^Ga]Ga-HA-DOTATATE are depicted in Fig. 2. The large prediction intervals were in accordance with the observed high interpatient variability in organ and tumor accumulation (Table 1). For [^68^Ga]Ga-DOTATATE, the predictions of spleen uptake were adequate, with 75%, 75% and 88% of all observations within the prediction interval for group 1, 2 and 3, respectively. Evaluation with primary tumor observations showed a slight overprediction by the model, since observations were within the prediction interval for 42.9% and 66.7% in groups 1 and 3, respectively (observations were not available for group 2). Some liver metastases observations were under- or overpredicted by the model, but, in general, predictions were acceptable with 50% and 73.9% of all predictions within population ranges in groups 2 and 3, respectively. Other metastases predictions showed high accuracy with 100% and 89.2% of all observations within population ranges for groups 2 and 3, respectively. For [^68^Ga]Ga-HA-DOTATATE, spleen uptake was underpredicted more often compared to [^68^Ga]Ga-DOTATATE, but precision of predictions was still acceptable (52.4%, 85.7% and 80.6% for groups 1, 2 and 3, respectively). In addition, all tumor compartment predictions showed high accuracy for [^68^Ga]Ga-HA-DOTATATE (83.3% (group 1), 66.7% (group 2), and 90.0% (group 3) for primary metastases, 100% (group 2) and 96.4% (group 3) for liver metastases, and 100% (group 1) and 86.2% (group 3) for other metastases), although model results showed both over- and underprediction for a few outliers.

**Table 3:** Fixed and fitted input parameters for the PBPK model, showing compound-specific parameter differences between [^68^Ga]Ga-DOTATATE and [^68^Ga]Ga-HA-DOTATATE, and system-specific parameter differences between the tumor compartments

Parameter	Fixed or fitted (*) value [range used in predictions]	
Compound-specific	[^68^Ga]Ga-DOTATATE	[^68^Ga]Ga-HA-DOTATATE
Molecular weight	1502.3 g/mol	1628.5 g/mol
Lipophilicity	− 3.69	− 3.12
*K*_D_	0.20 nmol/L	0.23 nmol/L *
*k*_off_	0.012 min^−1^	0.0071 min^−1^ *
*k*_int_	0.161 min^−1^	0.268 min^−1^
*k*_deg_	0.00012 min^−1^	0.00012 min^−1^
Fraction unbound	0.69	0.69

System-specific	Primary tumor	Liver metastases	Other metastases
Fraction vascular	0.21 [0.09–0.34]	0.17 [0.1–0.23]	0.075 [0.025–0.125]
Fraction interstitial	0.3	0.3	0.3
Blood flow	152 mL/min/100 g [53–252]	203 mL/min/100 g [139–363]	152 mL/min/100 g [53–252]
SSTR concentration (interstitial)	168 nmol/L	224 nmol/L	224 nmol/L

*K*_D_ Equilibrium dissociation constant; *k*_off_ Dissociation rate constant; *k*_int_ Internalization rate; *k*_deg_ Degradation rate; *SSTR* Somatostatin receptor

A sensitivity analysis was performed for the tumor compartments of the [^68^Ga]Ga-DOTATATE model and showed the importance of fraction vascular (sensitivity values of 0.655, 0.735 and 0.575 for primary tumor, liver metastases and other metastases, respectively) and the administered dose (sensitivity value of 1.03 for all tumor compartments) on tumor uptake (displayed as area under the curve (AUC)). All model parameters were included in the sensitivity analysis, which indicated that only seven parameters had a substantial impact (higher than 5% on tumor uptake, after a 10% adjustment of the parameter), meaning that model output was not highly reliant on input parameters. Results of the sensitivity values are shown in Table 4.

**Table 4:** Sensitivity analysis results for the tumor compartments with area under the concentration–time curve (AUC; 0–24 h) as output parameter

Compartment	Input parameter	Sensitivity value
Primary tumor	Fraction vascular of primary tumor	0.655
Primary tumor	[^68^Ga]Ga-DOTATATE dose (µg)	1.03
Liver metastases	[^68^Ga]Ga-DOTATATE dose (µg)	1.03
Liver metastases	Fraction vascular of liver metastases	0.735
Liver metastases	SA proportionality factor	0.527
Other metastases	[^68^Ga]Ga-DOTATATE dose (µg)	1.03
Other metastases	Fraction vascular of other metastases	0.575

Only sensitivity values < −0.5 or > 0.5 were reported

### DOTATATE versus HA-DOTATATE

**Fig. 3:**
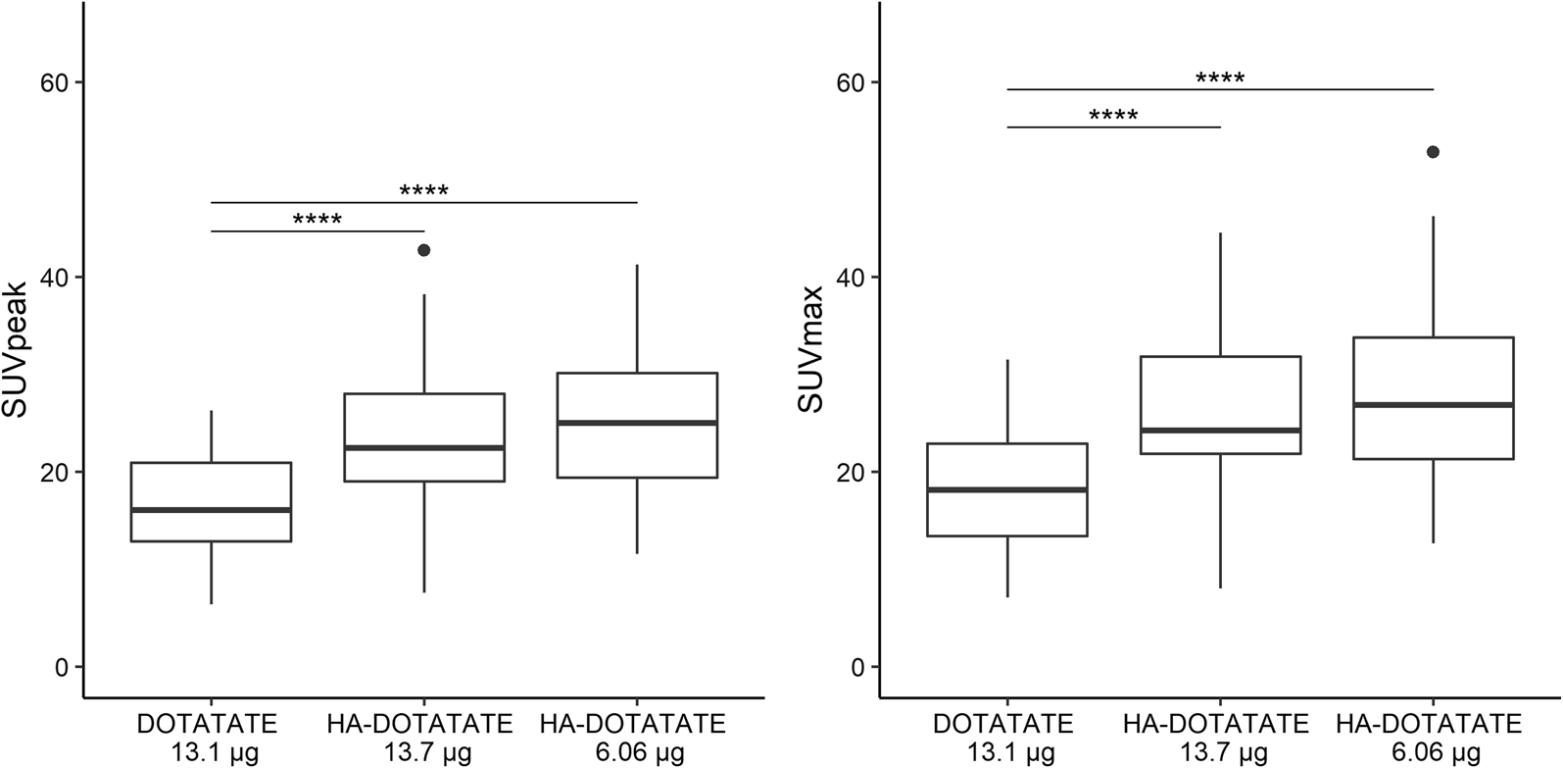
Comparisons of spleen uptake after PET/CT in patients receiving [^68^Ga]Ga-DOTATATE or [^68^Ga]Ga-HA-DOTATATE (low and high peptide amounts) showed a significant higher uptake for [^68^Ga]Ga-DOTATATE compared to [^68^Ga]Ga-HA-DOTATATE. In addition, peptide amount differences for [^68^Ga]Ga-HA-DOTATATE did not affect spleen SUV_peak_ and SUV_max_. Lines indicate significant uptake differences between two groups (*p ≤ 0.05, **p ≤ 0.01, ***p ≤ 0.001 and ****p ≤ 0.0001)

Results from PET/CT analyses showed a significant lower uptake (SUV_max_ and SUV_peak_) in spleen, liver, thyroid (*p* < 0.001 for all organs) and a significantly higher uptake in aorta (*p* < 0.001) for [^68^Ga]Ga-DOTATATE compared to [^68^Ga]Ga-HA-DOTATATE. Most likely, these dissimilarities in uptake were caused by different affinities for SSTR for both peptides. To exclude a potential additional effect of administered peptide amount on uptake differences between [^68^Ga]Ga-DOTATATE and [^68^Ga]Ga-HA-DOTATATE, a subanalysis was performed. Three groups were compared; patients receiving DOTATATE (mean peptide amount 13.1 µg), low amount HA-DOTATATE (mean peptide amount 6.06 µg) and high amount HA-DOTATATE (mean peptide amount 13.7 µg). Results of spleen SUV values of these three groups are shown in Fig. 3. A significant decrease in spleen uptake was observed for [^68^Ga]Ga-DOTATATE compared to [^68^Ga]Ga-HA-DOTATATE after administration of comparable peptide amounts, which implied that organ uptake differences were most probably caused by different affinity profiles. In PBPK model predictions, this was reflected in different values of *K*_D_ and *k*_off_ to describe differences in distribution profiles between both peptides. Moreover, different administered peptide amounts for HA-DOTATATE did not result in significant differences in spleen SUV_peak_ or SUV_max_ in this study population.

### Tumor sink effect

**Fig. 4:**
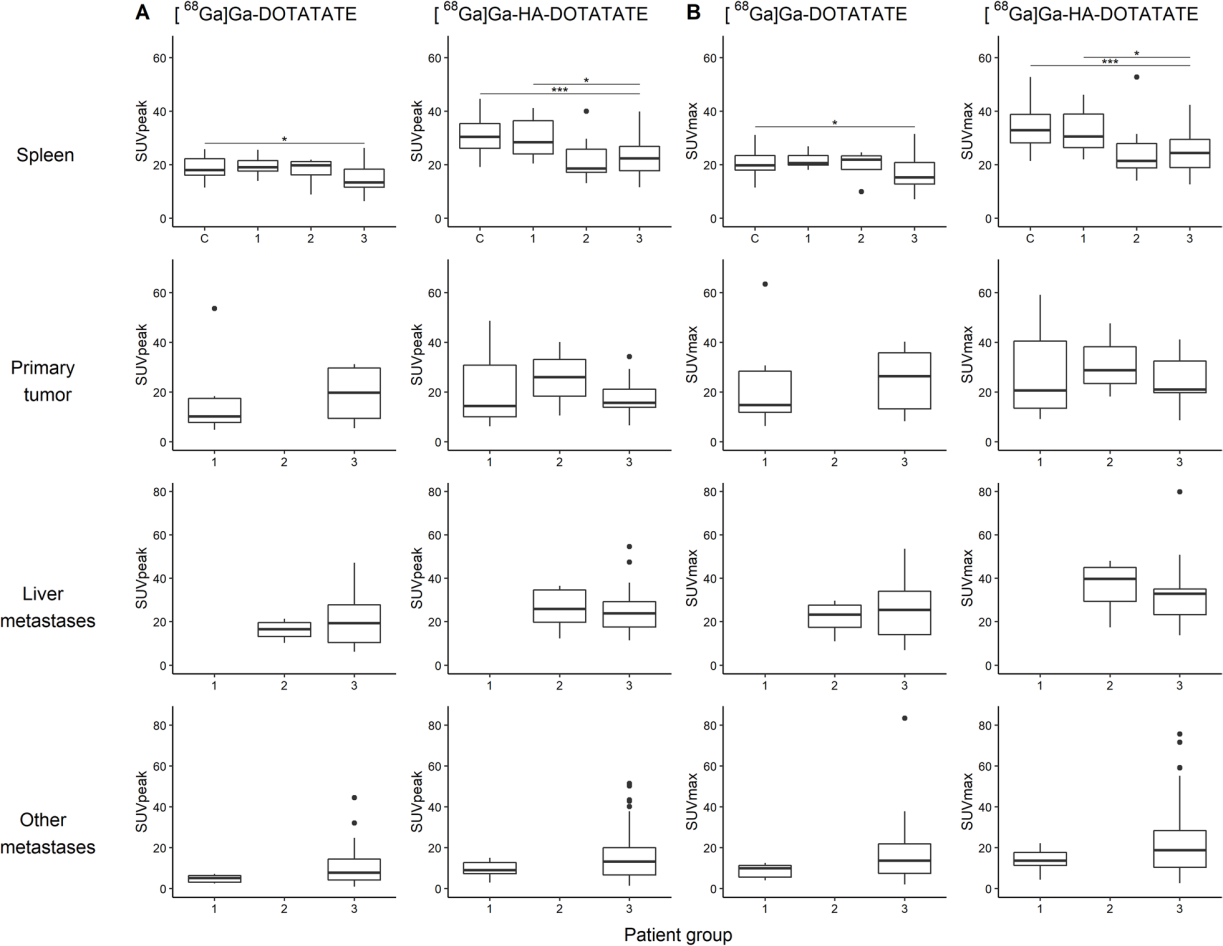
Spleen and tumor uptake (SUV_peak_ (**A**) and SUV_max_ (**B**)) of [^68^Ga]Ga-DOTATATE and [^68^Ga]Ga-HA-DOTATATE for: (C) control group, (1) limited disease, (2) liver-only metastases and (3) extensive metastases patients. Lines indicate significant uptake differences between two groups (*p ≤ 0.05, **p ≤ 0.01, ***p ≤ 0.001 and ****p ≤ 0.0001)

Organ uptake (SUV_peak_ and SUV_max_) was compared between the control group and all patient subgroups. A significant decrease in spleen uptake was observed for patients with extensive metastases compared to control subjects for both [^68^Ga]Ga-DOTATATE (SUV_peak_: 15.2 ± 5.68 vs. 18.85 ± 3.95, *p* = 0.0301 and SUV_max_: 17.0 ± 6.52 vs. 20.9 ± 4.20, *p* = 0.0290) and [^68^Ga]Ga-HA-DOTATATE (SUV_peak_: 22.9 ± 7.44 vs. 30.7 ± 6.86, *p* < 0.001 and SUV_max_: 25.0 ± 7.97 vs. 33.9 ± 7.53, *p* < 0.001). Moreover, for [^68^Ga]Ga-HA-DOTATATE a significant decrease in spleen uptake for this group compared to patients with only primary tumor and/or few metastases was noticed (SUV_peak_: 22.9 ± 7.44 vs. 29.4 ± 6.58, *p* = 0.0418 and SUV_max_: 25.0 ± 7.97 vs. 32.4 ± 7.39, *p* = 0.0304). This implied a probable tumor sink effect only for patients with extensive metastases. No significant differences in primary tumor and metastases uptake were observed between the patient groups. Results of spleen and tumor uptake differences between the groups are depicted in Fig. 4. Other organs (liver and thyroid) showed similar effects as for spleen (see Additional file 1: Fig. S1).

**Fig. 5:**
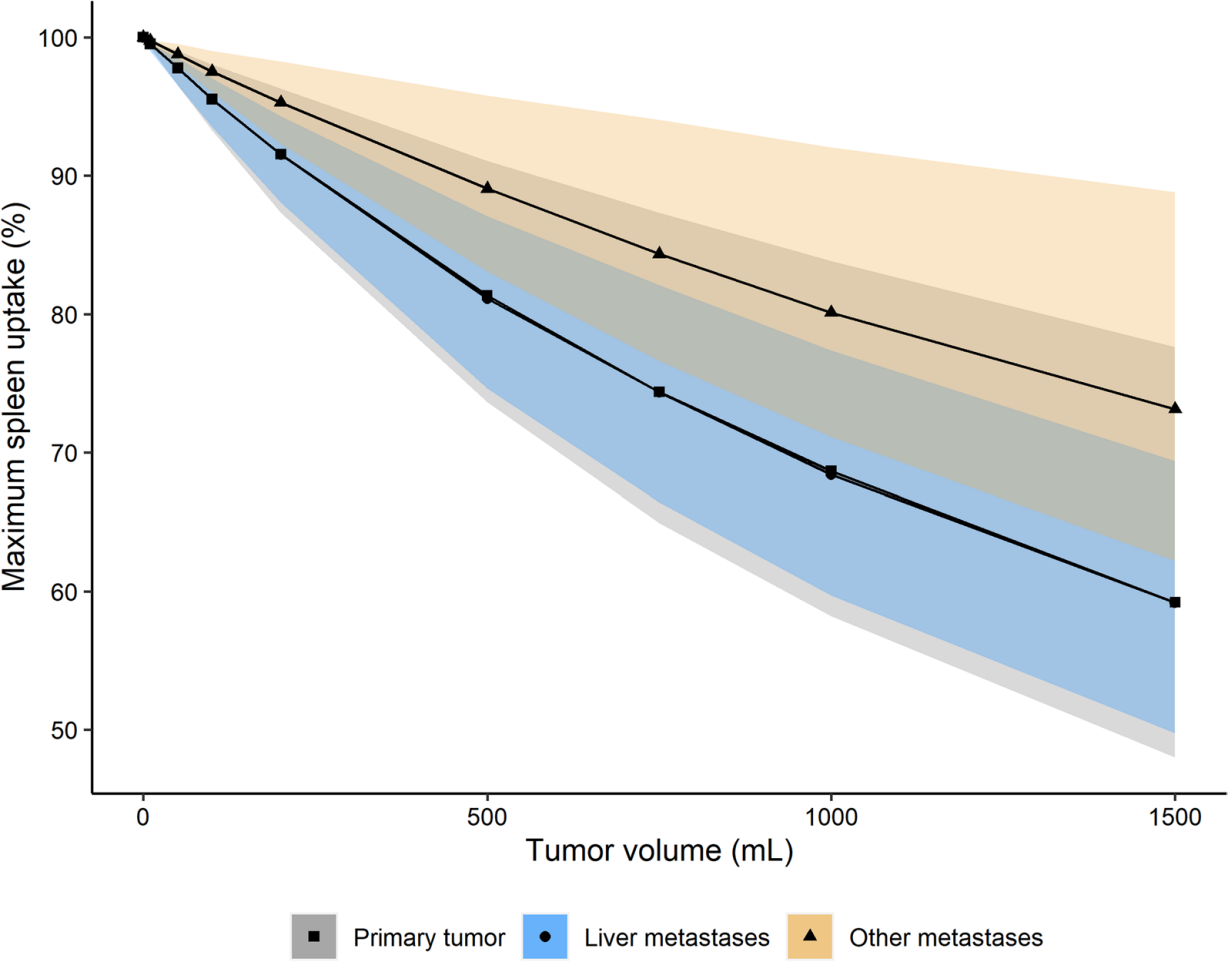
Predictions of tumor sink effect for each tumor compartment (points and lines) (where primary tumor and liver metastases predictions are overlapping) with ranges in predictions (shaded areas) based on inter-individual variability of tumor blood flow and tumor blood volume

Simulations, based on the developed PBPK model, with varying total tumor volumes of 0 to 1500 mL resulted in more detailed insights in the relevance of the tumor sink effect by comparing maximum accumulation concentrations (C_max_) in spleen. All tumor compartments and population ranges for tumor blood flow and blood volume were included in the simulations, to include possible tumor-specific effects and to reveal potential minimum and maximum ranges for the tumor sink effect for different tumor volumes. Predictions showed a decrease in C_max_ in spleen after increasing total tumor burden, with a maximum decrease of 41% for 1500 mL primary tumor volume. Predictions showed that a relevant tumor sink effect (> 20%) was only present in patients with an extensive tumor load above approximately 550 mL. However, at lower tumor volumes (< 200 mL, representing the majority of patients in our clinical population) only a modest decrease in spleen uptake was predicted (8.5% for primary tumors (range 3.7–12.7%)). Results of the tumor sink predictions are displayed in Fig. 5. This prediction of the existence of a tumor sink effect particularly in patients with high total tumor burden was comparable to the observed uptake data within the patient subgroups.

## Discussion

A PBPK model was developed to describe biodistribution and tumor uptake of [^68^Ga]Ga-DOTATATE and [^68^Ga]Ga-HA-DOTATATE in GEP-NET patients. Evaluation based on observed imaging data showed that these models adequately predicted peptide uptake in selected organs and tumors for patients with different tumor loads and location of lesions. A subanalysis showed that [^68^Ga]Ga-DOTATATE spleen uptake (main reference organ) was significantly lower compared to [^68^Ga]Ga-HA-DOTATATE, irrespective of the amount of peptide administered consistent with affinity differences. In addition, differences in administered peptide amount of [^68^Ga]Ga-HA-DOTATATE did not affect spleen uptake. Lastly, scan observations and predictions by the PBPK model showed that a clinically relevant tumor sink effect was only present in patients with an extensive tumor load.

### Patient population

In the current study, patients were divided based on their tumor load into three groups: (1) patients with primary tumors or with ≤ 2 metastases (limited disease), (2) patients with liver-only metastases and (3) patients with extensive metastases. Our classification roughly follows the work of Riihimaki et al*.* [53] and reflects the three main categories of patients. For this modeling study, it was important to have a simplified resemblance of a clinical population, which also provided a subgroups with comparable tumor burden. Lastly, liver metastases have a high incidence in metastatic NET and are known to have different uptake compared to other metastases and primary tumors [12, 53, 54]. The latter two arguments were both important factors for the identification of a potential tumor sink effect.

Because of the retrospective nature of this study, the total included patients differed between [^68^Ga]Ga-DOTATATE and [^68^Ga]Ga-HA-DOTATATE groups (*n* = 39 vs. *n* = 59, respectively). However, patient data were only used for model evaluation and we believe that our data were sufficient to evaluate biodistribution for both peptides in this population. Also, since parameter optimization was performed based on total patients rather than smaller patient groups, this difference in group size was not likely to affect model predictions. The control group was created from patients without apparent DOTATATE-positive lesions (i.e., signal intensity higher than local background) on diagnostic PET/CT. This group was assumed to only have physiological tracer accumulation, but actually consisted of patients that were referred with suspected GEP-NET or received treatment for prior NET. Still, we believe that this assumption did not influence our findings and model predictions.

### PBPK model

By calculating peptide concentrations based on measured radioactivity and administered specific activities, the assumption was made that peptide degradation did not occur during circulation. Intracellular degradation of peptides was described with a fixed degradation rate constant, but it was unlikely that this impacted our assumptions of observed data. Firstly because the rate constant is very small and thus degradation is limited, but also because most degradation occurs at points in time after the observed data.

Despite evidence that SSTR expression is highly variable between individuals [55], changing SSTR2 expression for tumors did not result in substantial different tumor uptake predictions. Results of the sensitivity analysis supported this finding (the sensitivity value of SSTR2 amount was 0.06 for primary tumor) and, therefore, SSTR2 expression on tumors was fixed in further analyses. Sensitivity analysis results also showed the importance of blood volume (fraction vascular) on tumor uptake. Tumor blood flow only slightly affected tumor uptake as shown by the sensitivity analysis (0.237 for primary tumor). However, it should be mentioned that a 10% change in tumor blood flow is not representative for the known inter-individual variability [42–45]. Manual adjustments of tumor blood flow based on reported literature variability values clearly affected tumor uptake and, therefore, these values were included as population ranges in the final PBPK tumor models.

Two main considerations should be pointed out about the model not being able to predict all data observations, especially for liver metastases and other metastases. Firstly, segmentation of tumor lesions was based on lesions with highest uptake and, therefore, were probably not representative for average expected tumor uptake. Secondly, interquartile ranges of tumor blood flow and blood volume were used to predict minimum and maximum uptake ranges. Data observations outside of these predicted ranges most probably represented patients with, for example, extensive tumor blood flow and blood volume.

### DOTATATE versus HA-DOTATATE

Higher organ uptake of [^68^Ga]Ga-HA-DOTATATE compared to [^68^Ga]Ga-DOTATATE was described previously, although these differences seemed on average minor and tumor uptake was found to be comparable for [^68^Ga]Ga-DOTATATE and [^68^Ga]Ga-HA-DOTATATE [5, 32]. Sensitivity analysis of the PBPK model showed that slight differences in chemical properties, such as molecular weight and lipophilicity, could not fully explain the observed organ uptake dissimilarities between the tracers. In the subanalysis regarding administered peptide amount, it was shown that this peptide amount did not cause the difference in spleen uptake. These findings together lead to the conclusion that differences in receptor affinity are the likely cause for increased organ uptake for HA-DOTATATE. This was taken into account in the PBPK model by the optimization of affinity (*K*_D_ and *k*_off_) of HA-DOTATATE to describe uptake differences between both peptides.

Apparently, the increased affinity of HA-DOTATATE did not affect (primary) tumor uptake, since primary tumor uptake was not significantly different between both peptides (Table 1). Results of the PBPK model were in agreement with these observations, because tumor blood flow and volume showed most effect on tumor uptake and not SSTR binding or expression of the receptor. In addition, the subanalysis showed that administered peptide amount did not alter spleen uptake (for HA-DOTATATE), probably indicating that receptor saturation was not likely to occur within these relatively low ranges of peptide amounts (< 20 µg).

### Tumor sink effect

To the best of our knowledge, this is the first study to compare organ uptake in patient groups with varying tumor burden. Scan analysis showed no significant effect of total tumor burden on organ uptake for groups 1 and 2 compared to the control group; thus, a tumor sink effect was not present within the range of observed total tumor burden in these patients. PBPK model predictions also showed a small and probably clinically irrelevant tumor sink effect for tumors with rather low-to-moderate volumes (which represents the majority of patients) [37]. To clarify, for patients with a median NET volume (approximately 65 mL) [37], a decrease in spleen uptake of ~ 3% was predicted. However, PBPK model predictions showed a clear decrease in spleen accumulation for patients with increasing total tumor loads (Fig. 5). Although variability in predictions was high, it was expected that liver metastases play an import part for patients with extensive NET volumes and predictions within the minimum decrease range in spleen uptake (based on high tumor load of other metastases with low tumor blood flow and blood volume) will be less likely to occur. When looking at the day-to-day variability in DOTATATE spleen accumulation, differences of approximately 15% can be observed according to previous work of Aalbersberg et al*.* [56]. Therefore, we believe that a tumor sink effect of > 20% may be considered clinically relevant and, based on the model predictions, this only occurs in patients with total tumor loads higher than ~ 550 mL. These predictions were in agreement with scan observations, since for patients with extensive metastases (group 3), a measurable tumor sink effect was indeed demonstrated, where a significant lower organ uptake (spleen, liver, thyroid) was observed compared to the control groups (Fig. 4 and Additional file 1: Fig. S1).

Personalizing therapy by increasing injected activity for patients with extensive tumor burden will probably result in greater radiation delivery to tumor sites, while not exceeding maximum organ doses. Such a similar individualized approach was suggested by Beauregard et al*.*; however, their observed decrease in organ uptake was caused by increased tumor sequestration or increasing body size [28]. Unfortunately, tumor volumes were not analyzed as potential cause of this effect and, therefore, results remain difficult to compare. Still, increasing activity doses for patients with high total tumors loads remains an interesting topic for future research. The characterization of the tumor sink effect as a disease-drug interaction will allow the further use of PBPK models to personalize dosing in the near future. However, based on these results, it seems that only a small part of all treated NET patients would benefit from individualized dosing based on tumor volume, since a relevant tumor sink effect was only predicted for patients with total tumor loads higher than ~ 550 mL. Regarding peptide selection, model predictions demonstrated only slight organ and tumor uptake differences for DOTATATE compared to HA-DOTATATE despite their differences in physicochemical parameters and receptor affinity, and thus there seems no clear preference of one peptide over the other. Future model predictions could help to select optimal dosing regimens to increase tumor lesion uptake while limiting organ uptake in patients receiving these radiolabeled SSAs for both diagnosis and therapy.

## Conclusion

The developed PBPK model could adequately predict organ and tumor uptake of [^68^Ga]Ga-DOTATATE and [^68^Ga]Ga-HA-DOTATATE in 39 and 59 GEP-NET patients, respectively. Variability in tumor blood flow and tumor blood volume appeared most important to predict tumor uptake population ranges, while SSTR2 expression differences seemed less relevant. Furthermore, differences in administered peptide amount of [^68^Ga]Ga-DOTATATE and [^68^Ga]Ga-HA-DOTATATE did not affect organ uptake and affinity differences appeared to be mainly responsible for the significant differences in organ uptake observed between both peptides. Lastly, a decrease of reference organ uptake was not observed and predicted for the majority of NET patients with low total tumor loads, but for patients with higher total tumor volumes (> 550 mL) a clinically relevant tumor sink effect was predicted. These developed PBPK models could be pivotal in, for example, finding optimal individualized doses, selecting ligands with regard to achieving optimal organ and tumor distribution ratios and providing a basis for translating uptake between theranostic tracers.

## Supplementary Information

**Additional file 1: Table S1.** Patient details for the subanalysis groups regarding GEP-NET patients receiving higher amounts of [^68^Ga]Ga-HA-DOTATATE. **Fig. S1.** Results of liver and thyroid uptake (SUV_peak_ (A) and SUV_max_ (B)) on PET/CT after administration of [^68^Ga]Ga-DOTATATE and [^68^Ga]Ga-HA-DOTATATE for patient groups; C control group, (1) limited disease, (2) liver-only metastases and (3) extensive metastases

## Data Availability

The datasets analyzed during the current study are available from the corresponding author on reasonable request.

## Abbreviations

AUC: Area under the curve
DOTATATE: DOTA-Tyr^3^-octreotate
^68^Ga: Gallium-68
GEP: Gastroenteropancreatic
GFR: Glomerular filtration rate
HA-DOTATATE: (High affinity) DOTA-iodo-Tyr^3^-octreotate
^177^Lu: Lutetium-177
NET: Neuroendocrine tumor
PBPK model: Physiologically based pharmacokinetic model
PK: Pharmacokinetic
SSA: Somatostatin analogue
SSTR2: Somatostatin receptor 2
SUV: Standardized uptake value
VOI: Volume of interest
