# Supplementary material for: A physiologically based pharmacokinetic model for [68Ga]Ga-(HA-)DOTATATE to predict whole-body distribution and tumor sink effects in GEP-NET patients
Source: EJNMMI Res. 2023 Feb 3;13:8. doi: 10.1186/s13550-023-00958-7 (PMC9898489; doi:10.1186/s13550-023-00958-7)
Supplement: Supplementary file 1 — Additional file 1: Table S1. Patient details for the subanalysis groups regarding GEP-NET patients receiving higher amounts of [68Ga]Ga-HA-DOTATATE. Fig. S1. Results of liver and thyroid uptake (SUVpeak (A) and SUVmax (B)) on PET/CT after administration of [68Ga]Ga-DOTATATE and [68Ga]Ga-HA-DOTATATE for patient groups; C control group, (1) limited disease, (2) liver-only metastases and (3) extensive metastases [file 13550_2023_958_MOESM1_ESM.docx]

**Supplementary Material**

**A Physiologically Based Pharmacokinetic Model for [^68^Ga]Ga-(HA-)DOTATATE to Predict Whole-Body Distribution and Tumor Sink Effects in GEP-NET patients**

Submitted to EJNMMI Research

Hinke Siebinga^1,2^, Berlinda J. de Wit-van der Veen^2^, Jos H. Beijnen^1^, Thomas P.C. Dorlo^1^, Alwin D.R. Huitema^1,3,4^, Jeroen J.M.A. Hendrikx^1,2^

1 Department of Pharmacy & Pharmacology, The Netherlands Cancer Institute, Amsterdam, The Netherlands

2 Department of Nuclear Medicine, The Netherlands Cancer Institute, Amsterdam, The Netherlands

3 Department of Clinical Pharmacy, University Medical Center Utrecht, Utrecht University, Utrecht, The Netherlands

4 Department of Pharmacology, Princess Máxima Center for Pediatric Oncology, Utrecht, The Netherlands

**Corresponding author:** Hinke Siebinga, Department of Pharmacy & Pharmacology and Department of Nuclear Medicine, The Netherlands Cancer Institute, Amsterdam, The Netherlands, h.siebinga@nki.nl

**Supplementary Table 1** – Patient details for the subanalysis groups regarding GEP-NET patients receiving higher amounts of [^68^Ga]Ga-HA-DOTATATE. Continuous variables are shown as mean ± standard deviation (range) and categorical variables as number (%).

|  | **[^68^Ga]Ga-HA-DOTATATE (n=59)** |
| --- | --- |
| **Sex**   - **Females** - **Males** | 23 (39%)  36 (61%) |
| **Age (years)** | 63 ± 12 (29 – 85) |
| **Weight (kg)** | 79.3 ± 17.0 (43 – 118) |
| **Height (cm)** | 175 ± 8.73 (153 – 193) |
| **Peptide amount administered (µg)** | 13.7 ± 3.34 (8.98 – 21.9) |
| **Radioactivity administered (MBq)** | 94.2 ± 8.60 (68.6 – 107) |
| **Injection-acquisition interval (minutes)** | 46 ± 5 (35 – 58) |
| **Primary tumor**   - **Pancreas** - **Small intestine** - **Colon** - **Rectum** - **Stomach** | 8 (14%)  37 (63%)  7 (12%)  5 (8%)  2 (3%) |
| **Tumor grade**   - **1** - **2** | 37 (63%)  22 (37%) |

**
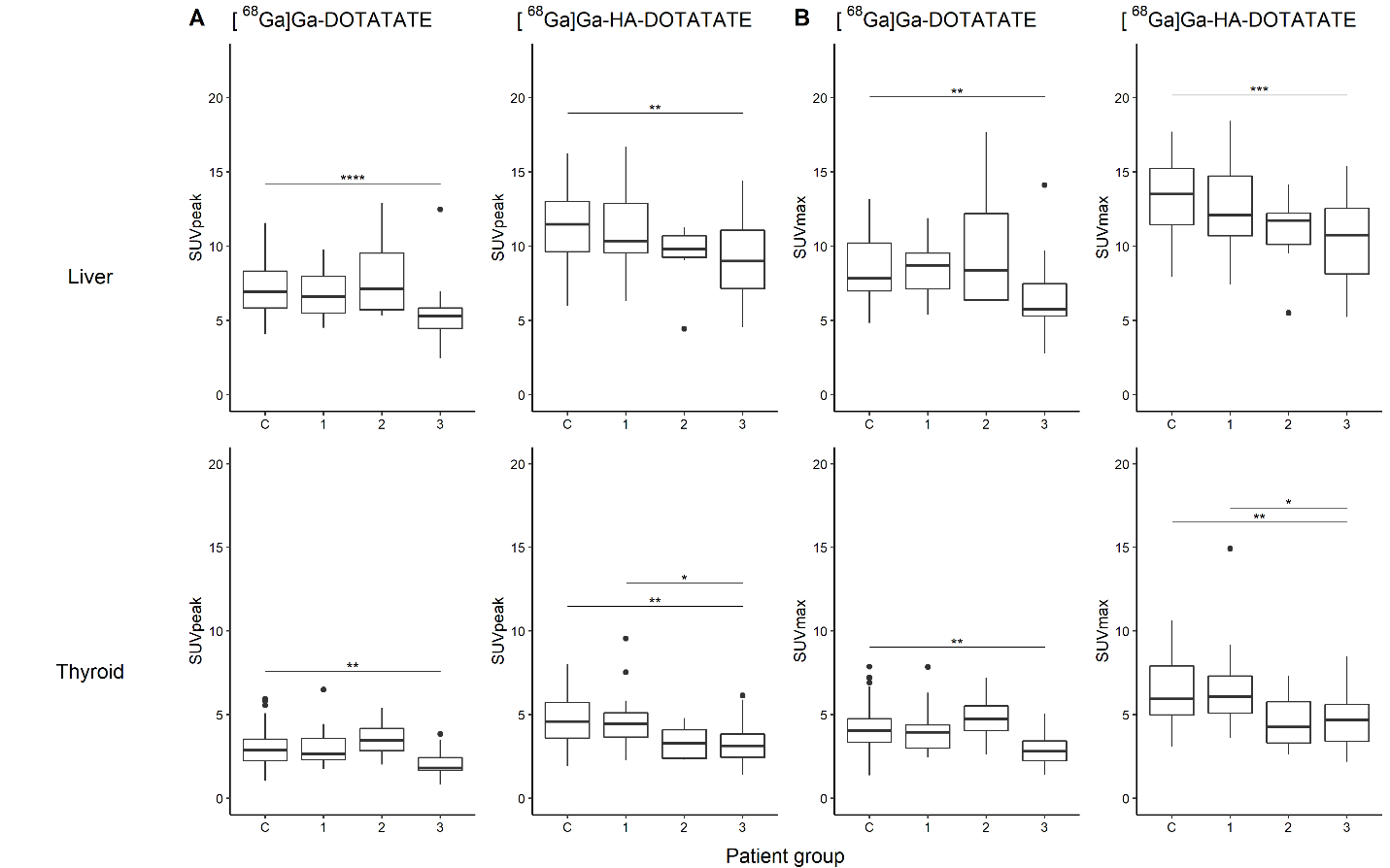
**

**Supplementary Figure 1** – Results of liver and thyroid uptake (SUV_peak_ (A) and SUV_max_ (B)) on PET/CT after administration of [^68^Ga]Ga-DOTATATE and [^68^Ga]Ga-HA-DOTATATE for patient groups; C) control group, 1) limited disease, 2) liver-only metastases and 3) extensive metastases. Lines indicate significant uptake differences between two groups (* p≤0.05, ** p≤0.01, *** p≤0.001 and **** p≤0.0001).
